# Efficient Removal of Methylene Blue from Aqueous Solutions Using a High Specific Surface Area Porous Carbon Derived from Soybean Dreg

**DOI:** 10.3390/ma14071754

**Published:** 2021-04-02

**Authors:** Zhiwei Ying, Lu Huang, Lili Ji, He Li, Xinqi Liu, Chi Zhang, Jian Zhang, Guofu Yi

**Affiliations:** 1 National Soybean Processing Industry Technology Innovation Center, Beijing Technology and Business University (BTBU), Beijing 100048, China; yingzhiwei0906@163.com (Z.Y.); huanglulu1119@163.com (L.H.); cauzhangchi@163.com (C.Z.); tsnpzhj@163.com (J.Z.); yiguofu0203@163.com (G.Y.); 2 Beijing Advanced Innovation Center for Food Nutrition and Human Health, Beijing Engineering and Technology Research Center of Food Additives, Beijing Technology and Business University (BTBU), Beijing 100048, China; 3 Institute of Innovation and Application, Zhejiang Ocean University, Zhoushan 316022, China; jll-gb@163.com

**Keywords:** soybean dreg, porous carbon, activation, adsorption, dye

## Abstract

Porous carbon material with high specific surface area was prepared from soybean dreg by a simple and effective two-step method (high temperature pyrolysis and activation). The structural characteristics of the synthesized carbon were evaluated by Brunauer–Emmett–Teller (BET), N_2_ adsorption/desorption measurements/techniques, an elemental analyzer (EA), scanning electron microscopy equipped with energy dispersive X-ray spectroscopy (SEM-EDS), transmission electron microscopy (TEM), an X-ray diffractometer (XRD), Raman spectroscopy (Raman), a Fourier transform infrared spectrometer (FTIR), and X-ray photoelectron spectroscopy (XPS). The specific surface area of SDB-6-K was 2786 m^2^ g^−1^, the pore volume was 2.316 cm^3^ g^−1^, and the average pore size was 3.326 nm. The high specific surface area and effective functional groups of carbon material promoted the adsorption of methylene blue. The maximum adsorption capacity of SDB-6-K to methylene blue was 2636 mg g^−1^ at 318 K. The adsorption kinetic and isotherm data were most suitable for pseudo-second-order and Langmuir equations. The results showed that the adsorbent had excellent adsorptive ability and had good practical application potential in the field of dye wastewater treatment in the future.

## 1. Introduction

Organic dyes are widely used in food, textile, papermaking, pharmaceuticals, plastics, and cosmetics industries, and the discharge of dye wastewater has also increased during the production and processing process as economies develop dramatically quickly [1]. Generally, dye wastewater contains a variety of organic compounds, including phenyl, amino, azo, etc., has the characteristics of high toxicity, deep color, difficult biodegradation, strong photolysis, and oxidation resistance [2], and has become one of the main harmful industrial wastewaters [3]. Among dyes, methylene blue is a representative compound of water-soluble dyes and is a cationic dye that is widely used. Its long-term exposure can cause vomiting, nausea, anemia, and hypertension. If dye wastewater cannot be treated properly and discharged directly, it will destroy the ecological environment and affect human health. Therefore, a prominent environmental protection problem has been how to effectively treat dye wastewater to reach the discharge standard [4]. Several common methods have been developed to remove these organic dyes, for instance, adsorption [5], chemical oxidation [6], photochemical degradation [7,8], and ultrasonic degradation [9], among which the adsorption method is one of the most valuable methods for research and application owing to its easy operation, environmental friendliness, low cost, lower energy consumption, superior stability, high efficiency, and so on [1,10].

For the past few years, carbon materials have been favored by researchers because of their unique internal structures, excellent performance, and extensive application prospects [11]. In the current research, carbon materials with different structural characteristics have been used as effective adsorbents to remove organic dyes, including activated carbon [12], mesoporous carbon [13], and carbon nanotubes (CNTs) [14], among which activated carbon has become one of the most concerned adsorbents for its structural characteristics and excellent adsorption capacity. However, researchers have been looking for low-cost adsorbents with promising applications to replace activated carbon, on account of its high cost and limited use [15]. Nowadays, agricultural waste material has become one of the research hotspots as a precursor of carbon materials in dye wastewater treatment, owning to its advantages of high carbon content, renewability, and low cost, and these materials, including coffee grounds [11], popcorn [16], pineapple crown leaves [17], grape pulp, wheat bran [18], waste mushroom matrix [19], and orange peel [20] have been prepared into various biomass carbon adsorbents for removing organic dye from aqueous solution.

Soybean is a major cash crop in China [21], the annual production of which is up to 16 million tons. Soybean dreg (SD) is a by-product of soybean protein and soybean oil separation or tofu and soybean milk processing, which is rich in dietary fiber and protein. However, to the best of our knowledge, soybean dreg has not been effectively utilized except for animal feed, plant fertilizer, or direct waste [22] because of its rough taste, high moisture content, difficult storage, and low energy content [23,24].

The aim of this work was to study organic dye adsorption performance of porous carbon material with high specific surface area obtained from soybean dreg by pyrolysis and KOH activation. The adsorbents were characterized by Brunauer–Emmett–Teller (BET), N_2_ adsorption/desorption measurements/techniques, an elemental analyzer (EA), scanning electron microscopy equipped with energy dispersive X-ray spectroscopy (SEM-EDS), transmission electron microscopy (TEM), an X-ray diffractometer (XRD), Raman spectroscopy (Raman), a Fourier transform infrared spectrometer (FTIR), and X-ray photoelectron spectroscopy (XPS). The adsorption kinetics and thermodynamics performances of as-prepared samples to remove methylene blue (MB) were investigated. Then the adsorption performances of single dye and mixed dyes on as-prepared sample were also studied.

## 2. Materials and Methods

### 2.1. Materials

SD was provided by Shandong Yuxin Biotechnology Co., Ltd., Shandong Province. The soybean dreg was dried at 105 °C for 48 h in a vacuum oven (GZX-9030 MBE, Shanghai Boxun Industrial Co., Ltd., Shanghai, China).

Commercial activated carbon (AC) was purchased from Chengde Xingyuan Activated Carbon Co. LTD. Methyl orange (MO) and crystal violet (CV) were purchased from Tianjin Fuchen Chemical Reagent Factory. Potassium hydroxide (KOH), methylene blue (MB), rhodamine B (RhB), and hydrochloric acid (HCl, 37 wt%) were purchased from Sinopharm Chemical Reagent Co., Ltd. (Shanghai, China) All chemical reagents were of analytical grade and used directly without further purification.

### 2.2. Preparation of Porous Carbon Materials

A total of 30 g of dried soybean dreg powder was carbonized in a tubular furnace under nitrogen flow (250 mL min^−1^) up to 500, 600, 700, and 800 °C, respectively, at a rate of 10 °C min^−1^, and the final constant temperature was held for 60 min.

The obtained pre-carbonized sample was mixed with KOH in the mass ratio (KOH: pre-carbonized materials = 4:1 (m/m)) and ground evenly. Then it was put into a corundum crucible. Under the condition of nitrogen flow rate (250 mL min^−1^), the mixture sample was activated to 800 °C in a tubular furnace, and the heating rate was 10 °C min^−1^. Finally, the constant temperature was maintained for 90 min. When the temperature dropped to room temperature, the as-prepared samples were placed in a 5 wt% HCl solution and continuously stirred for 24 h, then washed with a large amount of distilled water until the pH of the filtrate was about 7, and further dried at 105 °C for 24 h. The as-prepared samples were denoted as SDB-X-K (X = 5, 6, 7, and 8, referring to the pre-carbonization temperature of 500, 600, 700, and 800 °C, respectively).

### 2.3. Characterization of Porous Carbon Materials

The pore characteristics was performed at 77 K on a nitrogen adsorption apparatus (BET, Quantachrome Autosorb-iQ, Boynton Beach, FL, USA). The weight ratio of the elements C, H, O, N, and S were analyzed by an elemental analyzer (Elementar Vario EL III, Hanau, Germany). In order to observe the surface state of as-prepared samples, the images of microstructure and morphology were performed by a scanning electron microscope (SEM; JEOL JSM-6700F, Tokyo, Japan) equipped with energy dispersive X-ray spectroscopy (EDS), and a transmission electron microscope (TEM; JEOL JEM-2100F). The phase composition and crystal structure were recorded by X-ray diffraction (Rigaku Ultima IV, Tokyo, Japan) in the range of 10–80° and Raman spectroscopy (Raman, Bruker Optics SENTERRA, Ettlingen, Germany, excitation-beam wavelength: 532 nm). The surface properties were analyzed by means of X-ray photoelectron spectroscopy (XPS; Thermo Scientific Escalab 250Xi, Waltham, MA, USA). In this paper, the surface chemical functional groups were determined by Fourier transform infrared spectrometry (FTIR; SHIMADZU Type 2000, Tokyo, Japan).

### 2.4. Adsorption of Organic Dyes

A total of 0.025 g of as-prepared sample was added to each of three Erlenmeyer flasks with 25 mL of 1000, 2000, and 3000 mg L^−1^ MB aqueous solutions, which were placed in a water-bathing constant temperature vibrator (WE-3, Tianjin Ounuo Instrument Co., Ltd., Tianjin, China) at 150 rpm for 2 h at 298 K. After adsorption, solid-liquid separation was carried out by centrifugation at 4000 rpm for 10 min; then the supernatant was filtered by 0.22 um filter, and its adsorption value was determined using an ultraviolet spectrophotometer (UV-vis, Agilent Cary-60, Palo Alto, CA, USA) at 664 nm, which is the maximum adsorption wavelength of MB. The adsorption amount Q (mg g^−1^) and removal efficiency R (%) of MB were, respectively, calculated according to Equations (1) and (2).

(1) $$Q\left({\mathrm{mg}\;g}^{-1}\right)=\frac{\left(C_{0}-C\right)V}{m}$$

(2) $$R(\%)\;=\frac{C_{0}-C}{C_{0}}\times 100\%$$

where Q (mg g^−1^) is the amount of dye adsorbed on the prepared sample, C_0_ (mg L^−1^) is the initial dye solution concentration, C (mg L^−1^) is the concentration of dye solution after adsorption (mg L^−1^), V (L) is the volume of dye solution, m (g) is the mass of adsorbent used, and R (%) is the removal efficiency of dye.

### 2.5. Study on Adsorption Kinetics and Thermodynamics

In the kinetic experiments, 0.025 g SDB-6-K was used to adsorb 25 mL of each of 1000, 2000, and 3000 mg L^−1^ MB solutions, and the adsorption capacity of MB at 298 K at different time intervals of 0 to 120 min was determined.

In the isotherm experiments, 0.025 g SDB-6-K was used to adsorb 25 mL of each of 100, 500, 1000, 1500, 2000, 2500, 2750, 3000, 3250, and 3500 mg L^−1^ MB solutions, and the adsorption performance was carried out for 2 h at 298, 308, and 318 K.

### 2.6. Adsorption Capacities of Single Dye and Mixed Dyes on SDB-6-K

To further investigate the adsorption of SDB-6-K, the adsorption capacities of single dye and mixed dyes on SDB-6-K were analyzed. A total of 0.025 g SDB-6-K was used to adsorb 25 mL of 1000 mg L^−1^ CV, MO, RhB, and MB aqueous solutions, and quaternary mixed dyes aqueous solution (CV + MO + RhB + MB; each one was 250 mg L^−1^). Their adsorption performances were conducted for 2 h at 298 K. The adsorption values were measured at 580 nm, 465 nm, 554.1 nm, and 664 nm, respectively, which are the maximum adsorption wavelengths of CV, MO, RhB, and MB. The adsorption efficiency of commercial activated carbon (AC) for different dyes was used for comparison.

## 3. Results and Discussion

### 3.1. Characterization of the As-Prepared Samples

#### 3.1.1. Porous Characterization and Element Analysis

**Figure 1:**
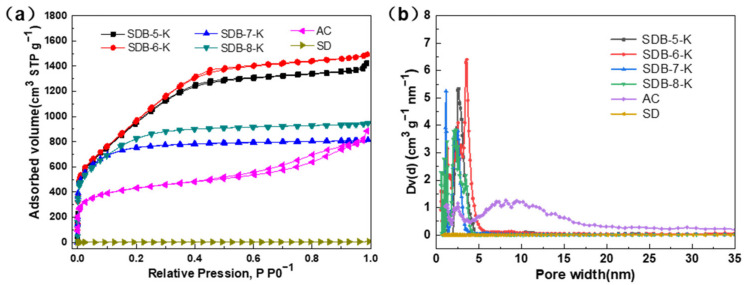
(**a**) N_2_ adsorption-desorption isotherms of SDBs-K, AC, and SD; (**b**) pore diameter distribution of SDBs-K, AC, and SD. AC is activated carbon; SD is soybean dreg.

The porous properties of SDBs-K, SD, and AC were measured by N_2_ adsorption/desorption isotherm. As the result show in Figure 1a, the raw material (SD) did not exist pores; however, after carbonization and activation, the as-prepared samples (SDBs-K) all exhibited type-I adsorption-desorption isotherms, which indicated the presence of micropores. A type-I adsorption-desorption isotherm is usually used to describe the micropore adsorption and monolayer adsorption, whereas SDB-5-K and SDB-6-K were relatively steep under low pressure, and micropore filling occurred. The linear diagram of the nitrogen isotherm showed pore condensation and H_2_ type hysteresis, indicating that the pore system was interconnected and shrunk. AC displayed a hysteresis loop at *p*/*p*_0_ > 0.5, which belonged to the type-IV adsorption-desorption isotherm, indicating the existence of micropores and mesopores. From these isotherms, it turned out that SDB-6-K had the highest specific surface area and pore volume compared to other adsorbents. Figure 1b shows that the pore characteristics of SDBs-K were mainly micropores, the majority concentrated in the range of 0.6–5.0 nm, and formed by activation of KOH [25] during high temperature pyrolysis and the KOH impregnated reaction. The framework of soybean dreg was etched to produce abundant micropores with the interaction between substances and the liberation of gas. In addition, when the activation temperature reached 800 °C, the carbon-reduced potassium metal was vaporized into gaseous potassium, and the potassium vapor entered the internal structure of the carbon and pushed into the interlayer of the carbon, leaving a large number of pores inside, thus forming a large specific surface area and rich pore structure [26].

As the data shown in Table 1 indicates, the SDBs-K prepared at different carbonization temperatures all had high specific surface areas, greater than 2500 m^2^ g^−1^. The surface areas of SDB-5-K, SDB-6-K, SDB-7-K, and SDB-8-K were 2611, 2786, 2604, and 2505 m^2^ g^−1^; the pore volumes were 2.206, 2.316, 1.465, and 1.263 cm^3^ g^−1^; and the average pore diameters were 3.378, 3.326, 2.339, and 1.941 nm, respectively. From the analysis of data results, the specific surface area, pore volume, and average pore diameter changed with the increase of carbonization temperature, which may be because the appropriate temperature would promote the formation of pores, and too high temperature would lead to the destruction of a large number of skeleton carbon and pore structure, so it was determined that SDB-6-K had the best pore structure.

The element content of SD and SDBs-K as shown in Table 2. After pyrolysis and KOH activation, the biochar yield of the as-prepared samples was over 11%, among which SDB-6-K reached the highest yield, up to 12.09%. Furthermore, the C contents of SDBs-K were all over 90%; however, the O, H, N, and S contents were all low, probably due to participation in the formation of gas. Combining the pore structure and biochar yield, SDB-6-K was selected as an optimum bio-adsorbent to remove organic dyes from aqueous solutions.

**Figure 2:**
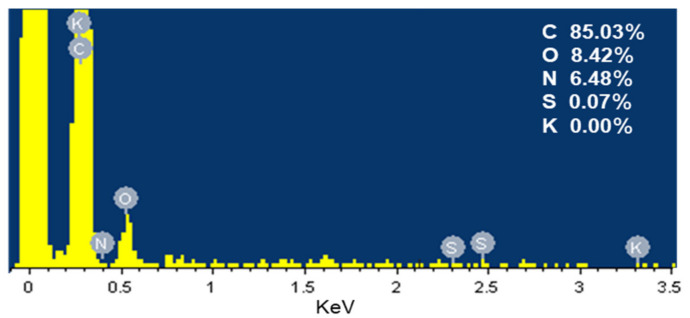
EDS spectrum of SDB-6-K.

EDS analysis in Figure 2 revealed that the SDB-6-K was composed of carbon (85.03%), oxygen (8.42%), nitrogen (6.48%), and sulfur (0.07%), which was consistent with the element analysis results, and the main components elements were C and O; no potassium was detected.

#### 3.1.2. Microstructure and Morphology Analysis

**Figure 3:**
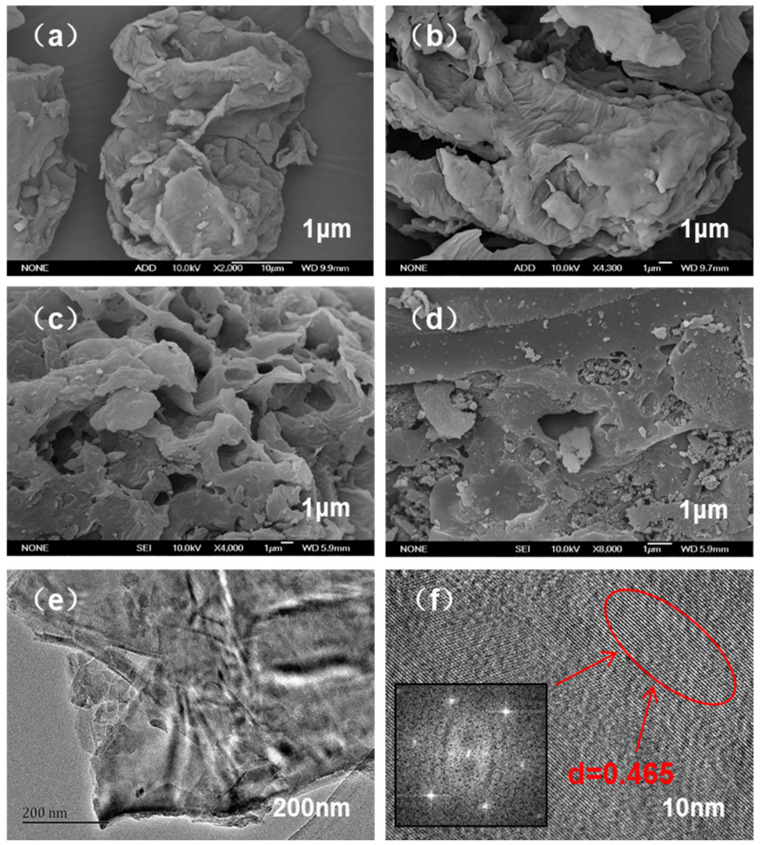
SEM images of (**a**) SD, (**b**) SDB-6, (**c**) SDB-6-K, and (**d**) AC; (**e**) TEM image of SDB-6-K; (**f**) HRTEM image of SDB-6-K.

According to the morphology of SD, SDB-6, SDB-6-K, and AC in Figure 3, the surface structure of SD was smooth, dense, and orderly and composed of many large blocks with few obvious pore structures [27]. After carbonization, abundant folds appeared on the surface of the SDB-6, and the pore structure was almost invisible. However, after KOH activation, the SDB-6-K possessed a honeycomb structure, with micropores uniformly arranged. Compared with SDB-6-K, the AC exhibited a dense and rough surface with different sizes of pores arranged.

Furthermore, the TEM images showed the internal apparent structure of SDB-6-K as a layered C structure of disordered stacks, and the lattice fringe was calculated from the fast Fourier transformation (FFT) of high resolution TEM (HRTEM) to confirm that the upper surface layer was a disordered C with a partial graphite domain, and the distinct lattice fringes with a distance of 0.465 nm, different from the graphite (002) plane (d = 0.34 nm) [28]. The graphene-like microcrystalline structure may be due to the high-temperature activation of KOH; the original closed hole was opened and reacted with disordered carbon atoms and heteroatoms to open the blocked hole and expose the microcrystalline surface.

#### 3.1.3. The Phase Composition and Crystal Structure Analysis

**Figure 4:**
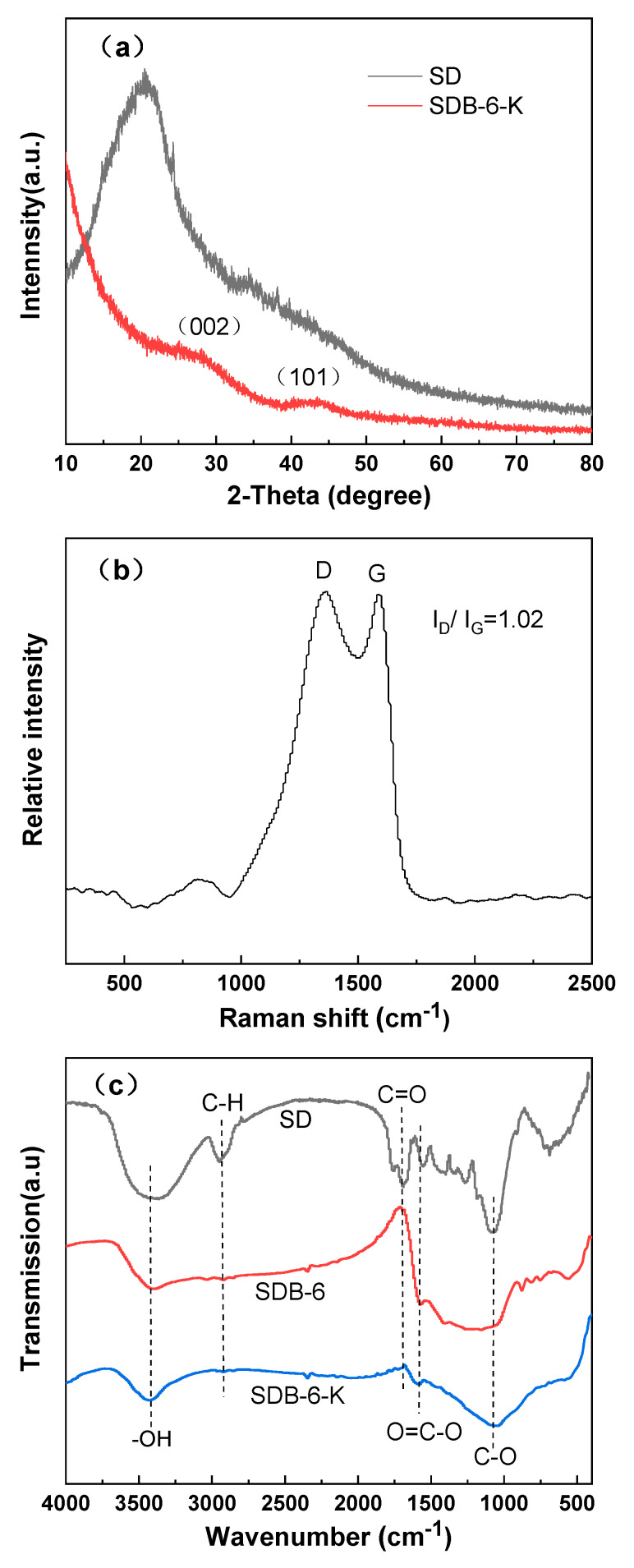
(**a**) XRD spectra of SD and SDB-6-K; (**b**) Raman spectra of SDB-6-K; (**c**) FTIR spectra of SD, SDB-6, and SDB-6-K.

The crystalline structures of SD and SDB-6-K were observed by XRD diffraction peaks. As shown in Figure 4a, a strong diffraction peak at 2θ = 20.7° appeared in the spectra of SD, corresponding to (002) crystal planes of cellulose [29]. After high temperature pyrolysis and KOH activation, there were two broad peaks at 2θ = 26.5°and 43.3°, which appeared in the spectra of SDB-6-K and corresponded to the (002) and (101) diffraction peaks, respectively, suggesting the presence of amorphous carbon [30], which was a crystal with the same structure as graphite; however, the lamellar structure formed by the hexagonal torus of carbon atom was disordered and irregular, with defects in crystal formation and small grains. Moreover, the low-intensity and broadened peaks indicated that the structure of SDB-6-K was highly disordered carbon [31].

There were two peaks near 1360 cm^−1^ and 1590 cm^−1^ in the Raman spectrum. As seen in Figure 4b, the D peak (1360 cm^−1^) was attributed to the disordered sp3-hybridized carbon, indicating the existence of disordered carbon structure, and the G peak (1590 cm^−1^) was related to the vibration of sp2-hybridized carbon atom in the graphite layer [32,33]. The ratio of I_D_/I_G_ represents the measurement of the disorder degree of graphite layers and the size of microcrystalline. Here, the I_D_/I_G_ ratio of SDB-6-K was 1.002, indicating that it had a certain graphitization structure, which was consistent with the TEM and XRD results.

The functional group compositions of as-prepared samples were investigated by FTIR, as seen in Figure 4c. As the sample contained an –OH functional group, the FTIR spectrum exhibited a peak at 3300–3500 cm^−1^ [34]. Due to the presence of a COO–functional group on the sample surface, the characteristic peak appeared at 1540–1650 cm^−1^ in the SD, SDB-6, and SDB-6-K spectra, and the spectra showed that the C–OH stretching vibration peak appeared at 1000–1300 cm^−1^ [16]. The peak at 2830–2930 cm^−1^ in the SD spectrum was ascribed to the C–H stretching vibration, followed by a peak located at 1680–1750 cm^−1^ attributed to the C=O stretching mode. From analysis of spectrum results, it could be demonstrated that most organic substrates had been removed by pyrolysis, and the as-prepared sample contained abundant functional groups, which greatly promoted the adsorption of organic dyes.

**Figure 5:**
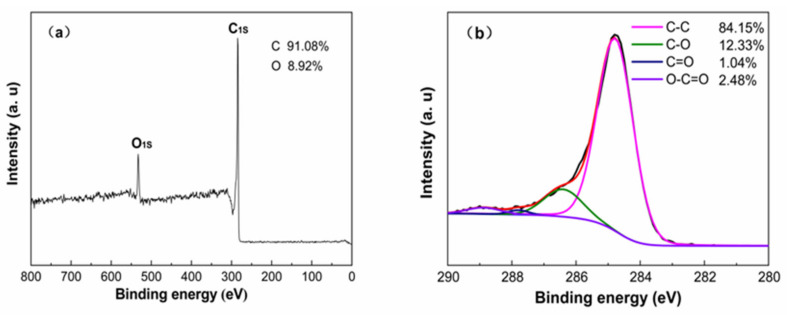
(**a**) XPS survey; (**b**) high-resolution spectra of C1s for SDB-6-K.

As can be observed from XPS images in Figure 5a, there were C1s (286.1 eV) and O1s (536.2 eV) peaks in the full X-ray photoelectron spectrum of SDB-6-K, containing a large amount of carbon (91.08%) and a small amount of oxygen elements (8.92%), which was consistent with the element content analysis results of EA. As shown in Figure 5b, the deconvolution of the C1s peak produced three individual peaks, representing C–O, carboxyl (COO–) and carbonyl (C=O) groups, respectively [33]. The surface of SDB-6-K had rich active oxygen-containing functional groups, and the exposure of defects provided a wealth of active sites; this result was consistent with that observed by FTIR spectroscopy [35].

### 3.2. Study of Adsorption Performance of Organic Dyes

#### 3.2.1. Adsorption Kinetics Study

Adsorption kinetics is critical to analyze adsorption mechanism. In order to elucidate the adsorption behavior of SDB-6-K on MB, two kinetic models were applied to analyze the adsorption mechanism: pseudo-first-order [36], and pseudo-second-order [37]. The formula is as follows:

(3) $${\ln(q}_{e}{-q}_{t}{)=\mathrm{lnq}}_{e}{-k}_{1}t$$

(4) $$\frac{t}{q_{t}}=\frac{1}{k_{2}q_{e}{}^{2}}+\frac{t}{q_{e}}$$

where q_e_ (mg g^−1^) is the adsorption amount of dye at equilibrium and q_t_ (mg g^−1^) is the adsorption amount of dye at time t (min); k_1_ (min^−1^) and k_2_ (g mg^−1^ min^−1^) are the rate constants from the two kinetic models, respectively.

**Figure 6:**
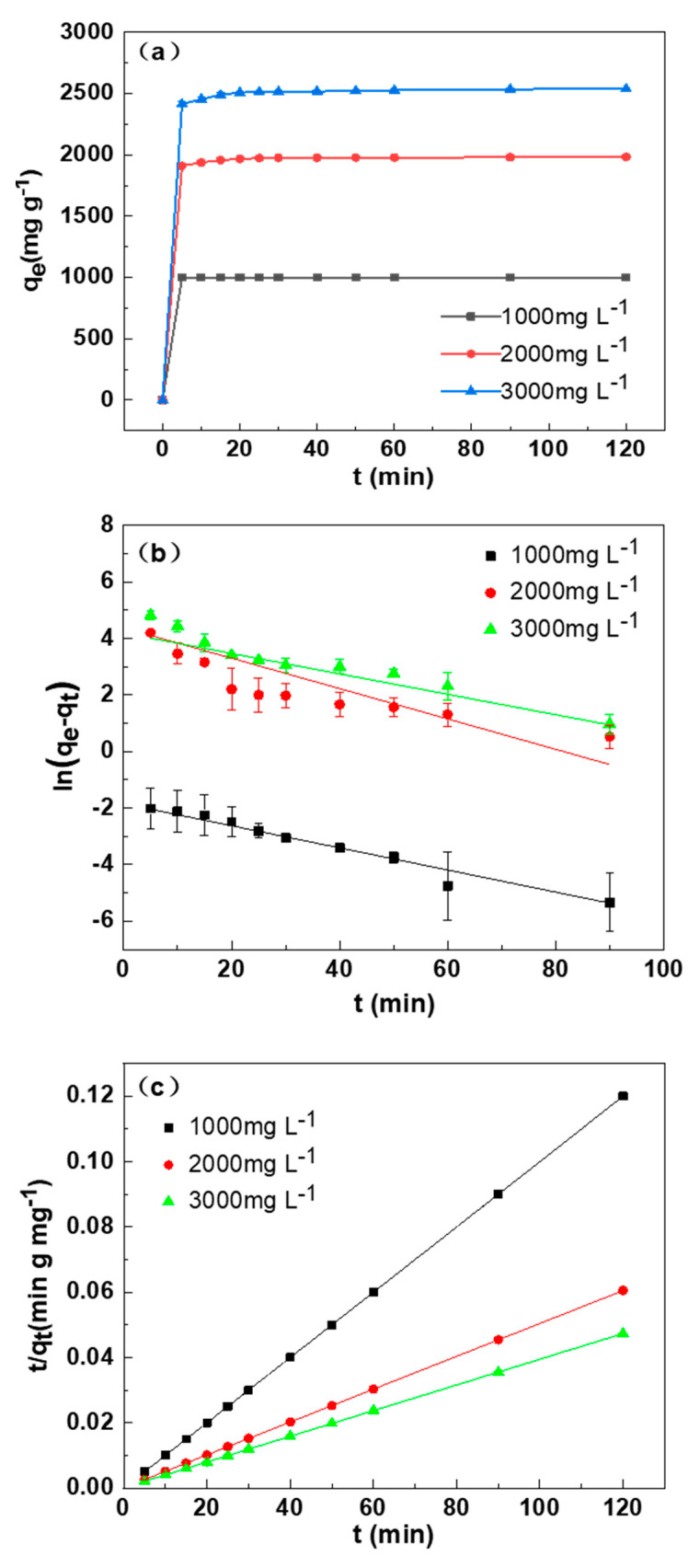
(**a**) Effect of the contact time on methylene blue (MB) adsorption by SDB-6-K. (**b**) The pseudo-first-order and (**c**) pseudo-second-order for the MB adsorption by SDB-6-K. Conditions: T = 298 K; m = 0.025 g; V = 25 mL; C_0_ = 1000, 2000, 3000 mg L^−1^.

Figure 6a indicated the adsorption capacity of SDB-6-K to different concentrations of MB at different adsorption times. With the extension of adsorption time, the adsorption efficiency increased until the adsorption equilibrium was reached. Furthermore, the maximum absorption amounts Q (mg g^−1^) of 1000, 2000, and 3000 mg L^−1^ MB were 999.7, 1982, and 2538 mg g^−1^, and removal efficiencies R (%) were 99.97%, 99.10%, and 84.60%, respectively. The adsorption efficiency of SDB-6-K on MB increased rapidly within 0.5 h and reached the adsorption equilibrium within 30 min, which may have been caused by the existence of effective binding sites and abundant microporous structures on the surface of SDB-6-K. With adsorption time extension, the adsorption process reached balance, and the adsorption sites reached saturation, so the adsorption capacity was maintained in equilibrium.

Figure 6b,c displays the MB experimental data and the fitting of the two kinetics models, and the two kinetics model fitting results of SDB-6-K adsorption of MB are summarized in Table 3. The results showed that the pseudo-second-order model had significantly higher R^2^ (coefficient of determination) values (>0.9999) in the three concentrations of MB than the pseudo-first-order model, suggesting that the adsorption of MB on SDB-6-K was also related to chemical action. Thus, the pseudo-second-order model was the optimum model to explain the adsorption of MB using SDB-6-K, being used to calculate the adsorption amounts at equilibrium of 1000, 2000, and 3000 mg L^−1^ MB on SDB-6-K, which were 1000, 1985, and 2540 mg g^−1^, respectively, in good consistency with experimental data (999.7, 1982, and 2538 mg g^−1^), which indicated an effective adsorption of MB by SDB-6-K. This may be attributed to the high surface area and abundant functional groups, which promoted the rapid diffusion of organic dye molecules.

#### 3.2.2. Adsorption Isotherms Study

In order to explain the adsorption mechanism of SDB-6-K, Langmuir [38] and Freundlich [39] isotherm models were employed to fit the adsorption isotherm data.

The Langmuir isotherm model assumes that monolayer adsorption occurs on the limited active sites on the surface of the adsorbent, the adsorption capacity between the adsorption sites is the same, and there is no interaction between the adsorbed molecules. The Langmuir equation can be expressed as

(5) $$\frac{C_{e}}{q_{e}}=\frac{C_{e}}{q_{m}}+\frac{1}{k_{L}q_{m}}$$

The Freundlich isotherm model assumes multilayer adsorption due to uneven adsorption heat distributed on the uneven surface of the adsorption material. The Freundlich equation can be expressed as

(6) $${\mathrm{lnq}}_{e}={\mathrm{lnk}}_{F}+\frac{1}{n_{F}}{\mathrm{lnC}}_{e}$$

where C_e_ (mg g^−1^) and q_e_ (mg g^−1^) are the concentrations of dye in solution and in adsorbent at the adsorption equilibrium, respectively. q_m_ (mg g^−1^) is the maximum adsorption capacity of dye, k_L_ (L mg^−1^) is the Langmuir isotherm constant, and k_F_ (mg g^−1^) is the Freundlich isotherm constant.

**Figure 7:**
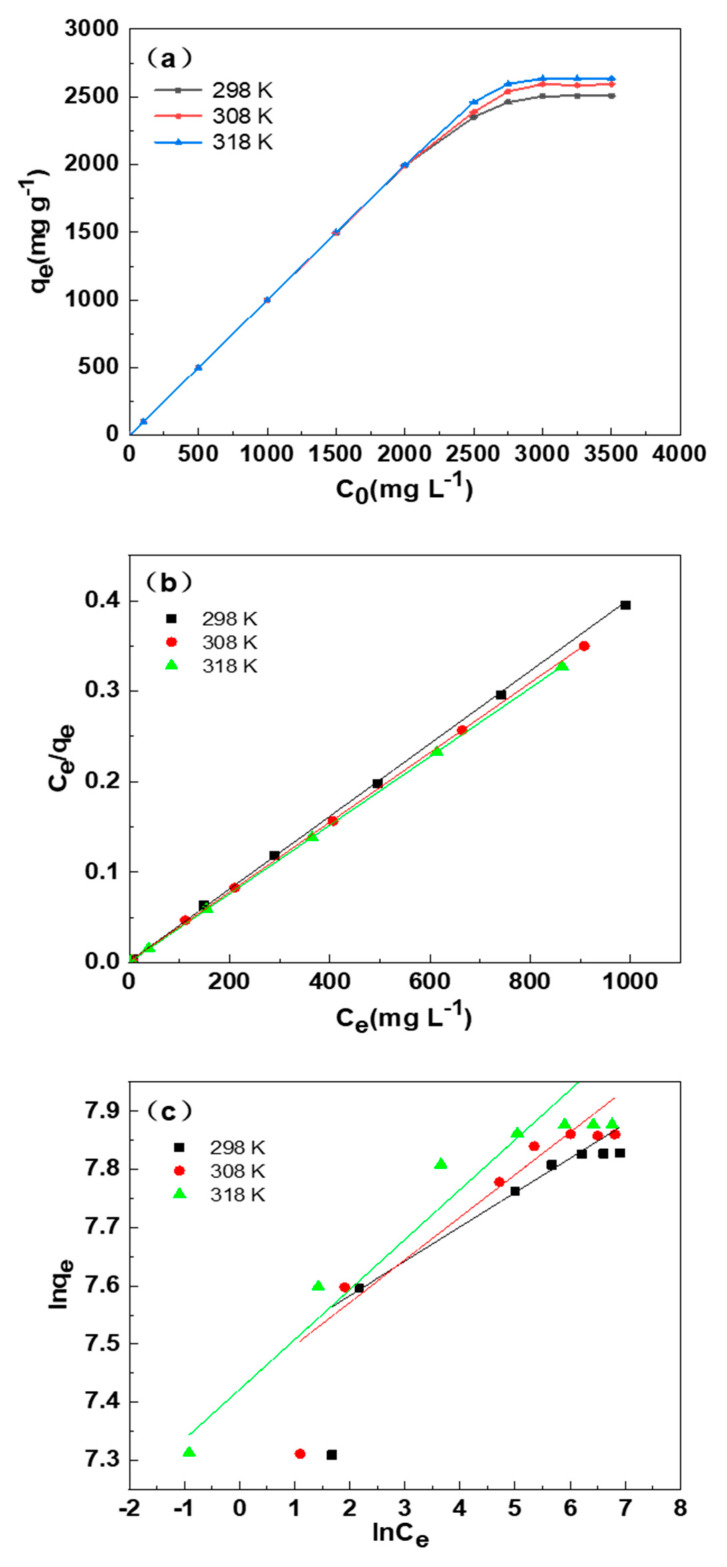
(**a**) Effect of dye initial concentration and temperature on the adsorption performance. (**b**) The Langmuir isotherms and (**c**) Freundlich isotherms for the MB adsorption by SDB-6-K. Conditions: T = 298, 308, 318 K; m = 0.025 g; V = 25 mL; C_0_ = 3000 mg L^−1^.

The adsorption isotherm of SDB-6-K for MB at different initial concentrations of MB and temperatures are illustrated in Figure 7a. It could be observed that the saturation adsorption capacity first increased sharply and then kept constant with the rising of the initial concentration of MB. Moreover, the temperature had no significant effect on the adsorption capacity when the initial concentration of MB was lower (2000 mg L^−1^), while the higher the temperature, the greater the adsorption capacity when the initial concentration of MB was more than 2000 mg L^−^^1^, which was indicating that the physical adsorption was dominant at lower initial concentration of MB, and the chemical adsorption was the main fashion at higher concentration.

Figure 7b,c indicates the fitting of experimental data with the two isotherm models, and the parameters and correlation coefficients of the two models are observed in Table 4. There were higher R^2^ values (0.9994, 0.9999, and 1.00) at 298 K, 308 K, and 318 K, respectively, in the Langmuir isotherm model, compared with those in the Freundlich isotherm model, and thus the Freundlich equation was more suitable for describing the adsorption of MB on SDB-6-K, indicating that this adsorption process was single-layer adsorption.

#### 3.2.3. Adsorption Thermodynamics Study

For the purpose of interpreting the thermodynamic behavior of MB adsorption for SDB-6-K, thermodynamic parameters were calculated using the following standard thermodynamic equations [40]:

(7) $${\mathrm{lnK}}_{c}=\frac{-\Delta H}{\mathrm{RT}}+\frac{\Delta S}{R}$$

(8) $$\Delta G=-{\mathrm{RTlnK}}_{C}$$

(9) $$K_{C}=\frac{q_{e}}{C_{e}}$$

where K_c_ is the equilibrium constant, ΔH (kJ mol^−1^) is the enthalpy change, ΔS (J K^−1^ mol^−1^) is the entropy change, ΔG (kJ mol^−1^) is the free energy change, R (8.314 J K^−1^ mol^−1^) is the universal gas constant, and T (K) is the system temperature.

Table 5 provides the thermodynamic parameters. The values of ΔH and ΔS can be obtained by calculating the slope and the intercept of the linear plot of ln Kc and 1/T. Both ΔH and ΔS were positive values, indicating that the adsorption reaction was endothermic, and the randomness of the solid-liquid interface during the adsorption process increased [41]. In addition, the ΔH values of the SDB-6-K were greater than zero, indicating that MB adsorption onto the SDB-6-K was an endothermic reaction. The ΔG values were negative, indicating that the MB adsorption on SDB-6-K was spontaneous adsorption, and it became more favorable at high adsorption temperatures. The positive ΔS confirmed the considerable interactions between the SDB-6-K functional sites and MB molecules.

In sum, the as-prepared sample (SDB-6-K) derived from soybean dreg has superior removal performance for organic dyes, which may be attributed to its abundant porous structure and high specific surface area. The high adsorption capacity of SDB-6-K may also be due to the abundant functional groups on its surface.

#### 3.2.4. Adsorption Performances of Single Dye and Mixed Dyes on SDB-6-K

**Figure 8:**
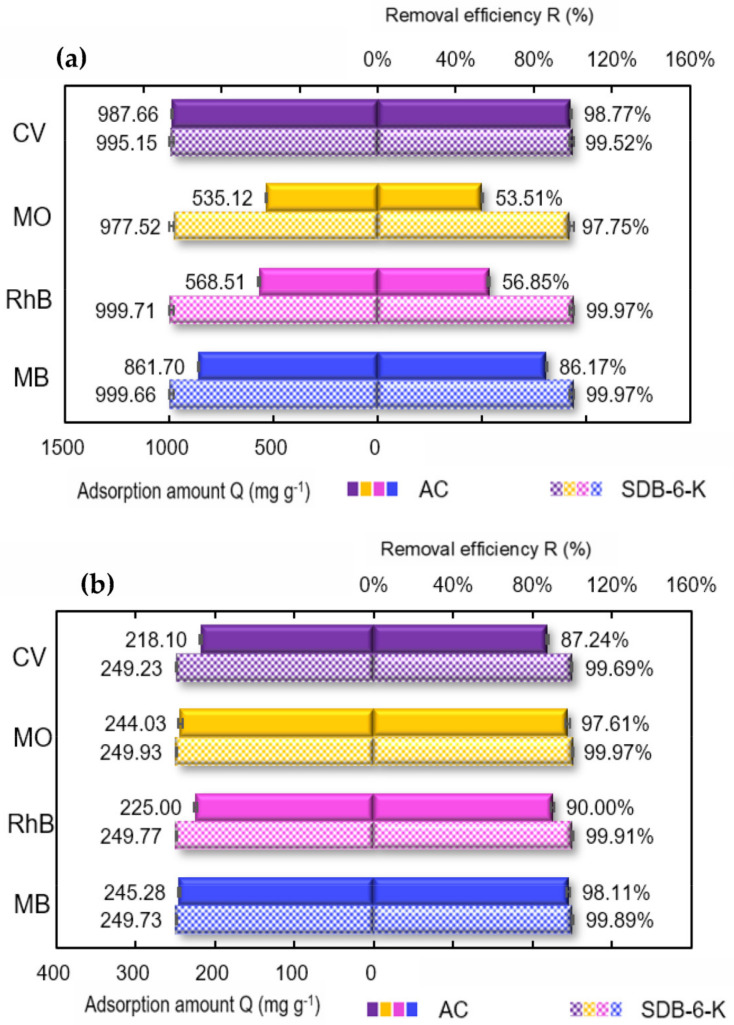
(**a**) Adsorption of different single dyes by SDB-6-K and AC; (**b**) adsorption of mixture dyes by SDB-6-K and AC. Conditions: (**a**) 1000 mg L^−1^ CV/MO/RhB/MB; (**b**) MB+RhB+MO+CV, each is 250 mg L^−1^; T = 298 K; m = 0.025 g; V = 25 mL; t = 2 h. SDB-6-K and AC. Conditions: (**a**) 1000 mg L^−1^ CV/MO/RhB/MB; (**b**) MB + RhB + MO + CV, each is 250 mg L^−1^; T = 298 K; m = 0.025 g; V = 25 mL; t = 2 h.

Considering the practical applications of the adsorbent, the adsorption performances for different single dyes and mixed dyes on SDB-6-K were investigated, and commercial activated carbon (AC) was used for comparison. As illustrated in Figure 8a, SDB-6-K had high adsorption efficiencies for four single dyes, and the adsorption amounts Q (mg g^−1^) of CV, MO, RhB, and MB were 995.15, 977.52, 999.71, and 999.66 mg g^−1^, respectively, and the removal efficiency R (%) was 99.52, 97.75, 99.97, and 99.97%, respectively. Compared with a single dye, SDB-6-K also had higher adsorption capacity for mixed dyes, as illustrated in Figure 8b, and the adsorption amounts Q (mg g^−1^) of CV, MO, RhB, and MB were 249.23, 249.93, 249.77, and 249.73 mg g^−1^, and the removal efficiency R (%) was 99.69, 99.97, 99.91, and 99.89%, respectively. It could be demonstrated that SDB-6-K exhibited higher adsorption efficiency for different single dyes and mixed dyes than AC, and has enormous potential in the application of dye wastewater treatment. In addition, compared with the maximum adsorption capacities of MB using the adsorbents derived from different sources previously reported, as shown in Table 6, it could be seen that the as-prepared SDB-6-K exhibited better adsorption performance of MB compared with the other adsorbents, owing to its graphene-like structure, large specific surface area, and developed porosity.

## 4. Conclusions

The porous carbon prepared by the two-step method (high temperature pyrolysis and KOH activation method) using soybean dreg as a raw material had a high specific surface area, a developed porosity, and suitable pore diameter and pore volume. The maximum adsorption amount of SDB-6-K to MB reached 2636 mg·g^−1^ at 318 K. It had excellent adsorption capacity for different single dyes (CV, MO, RhB, MB) and mixed dyes (CV + MO + RhB + MB). In general, SDB-6-K has good adsorption capacity, which has good practical application potential in the field of dye wastewater treatment in the future.

## Figures and Tables

**Table 1:** Specific surface area, pore volume, and average pore diameter of SDBs-K, AC, and SD.

Sample	Specific Surface Area (m^2^ g^−1^)	Pore Volume (cm^3^ g^−1^)	Average Pore Diameter (nm)
SDB-5-K	2611	2.206	3.378
SDB-6-K	2786	2.316	3.326
SDB-7-K	2604	1.465	2.339
SDB-8-K	2505	1.263	1.941
AC	1502	1.373	3.665
SD	7.130	0.011	6.110

**Table 2:** The yield and the elemental analysis of SDBs-K.

Sample	Yield (%)	C (%)	O (%)	H (%)	N (%)	S (%)
SDB-5-K	11.04%	91.96	6.40	1.149	0.335	0.317
SDB-6-K	12.09%	91.80	7.26	1.072	0.371	0.424
SDB-7-K	12.03%	91.14	7.58	0.842	0.351	0.272
SDB-8-K	11.89%	90.44	8.56	0.688	0.317	0.227

**Table 3:** Kinetic model parameters for the adsorption of MB onto SDB-6-K at different dye concentrations.

Sample	C_0_ (mg L^−1^)	q_e_, Experiment (mg g^−1^)	Pseudo-First-Order			Pseudo-Second-Order		
			q_e_, Calculated (mg g^−1^)	k_1_ (min^−1^)	R^2^	q_e_, Calculated (mg g^−1^)	k_2_ (g mg^−1^ min^−1^)	R^2^
SBD-6-K	1000	999.7	0.158	0.0430	0.9563	1000	0.9820	1.00
	2000	1982	79.17	0.0537	0.8837	1985	0.0028	1.00
	3000	2538	65.91	0.0361	0.7103	2540	0.0014	1.00

**Table 4:** Isotherm model parameters for the adsorption of MB onto SDB-6-K at different temperatures.

Sample	T/K	Langmuir			Freundlich		
		q_m_ (mg g^−1^)	K_L_ (L mg^−1^)	R^2^	n_F_	K_F_	R^2^
SDB-6-K	298	2488	0.39	0.9994	16.95	1746	0.8493
	308	2595	0.37	0.9999	13.67	1678	0.8866
	318	2639	1.04	1.00	11.65	1672	0.9121

**Table 5:** Thermodynamic parameters for the adsorption of MB onto SDB-6-K at different temperatures.

T	ΔG (kJ mol^−1^)	ΔH (kJ mol^−1^)	ΔS (J K^−1^ mol^−1^)
298	−4.0314	15.7828	66.4906
308	−4.6963		
318	−5.3612		

**Table 6:** Comparation of the maximum MB adsorption capacities of adsorbents prepared from different sources.

Adsorbent	BET (m^2^ g^−1^)	q_max_ (mg g^−1^)	q_max_/BET (mg m^−2^)	Reference
Banana peel	2086	385.12	0.185	[42]
Bean dreg	1738.95	434.78	0.250	[43]
Seaweed	926.39	512.67	0.553	[44]
Coffee grounds	1910	653.6	0.342	[11]
Coconut shells	876.14	200	0.228	[45]
Bamboo	1896	454.20	0.240	[46]
Rattan	1135	359	0.316	[47]
Fish scales	1867.67	184.40	0.099	[48]
Soybean dreg	2786	2636	0.946	This study

## Data Availability

Data sharing is not applicable to this article.
